# Effect of a Semirigid Ankle Brace on the In Vivo Kinematics of Patients with Functional Ankle Instability during the Stance Phase of Walking

**DOI:** 10.1155/2019/4398469

**Published:** 2019-04-08

**Authors:** Gonghao Zhang, Shengxuan Cao, Chen Wang, Xin Ma, Xu Wang, Jiazhang Huang, Chao Zhang

**Affiliations:** Department of Orthopedics, Huashan Hospital, Fudan University, Shanghai 200040, China

## Abstract

An ankle brace is commonly used by patients after they suffer from initial ankle sprains, reducing the incidents of recurrent sprain or limiting laxity in joints with functional ankle instability (FAI). However, whether the application of a semirigid ankle brace can improve the abnormal ankle gait kinematics of patients with FAI remains unknown. This study aimed to determine the effect of a semirigid ankle brace on the gait kinematics of ankle joints through 3D-2D fluoroscopy image registration. A total of 8 subjects with FAI (3 males and 5 females, 10 feet) as FAI group and 10 subjects without FAI (6 males and 4 females, 10 feet) as control group were enrolled in this study. Three-dimensional bone models created from computed tomography images were matched to fluoroscopic images to compute the 6 degrees of freedom (DOF) talocrural, subtalar, and ankle joints complex kinematics for control and FAI group with or without brace during the stance phase of walking. FAI patients had significantly less ROMs in inversion/eversion rotation of the talocrural and subtalar joint after wearing semirigid ankle brace. Laxity was observed in most of the displacements of the talocrural and subtalar joints in FAI group. The brace partly altered the ankle joints movement in opposite directions, especially joint rotation, and restricted the talocrural and subtalar joints in the dorsiflexion position during the touch down phase of walking.

## 1. Introduction

The most common injury encountered in sports is ankle sprain [1], particularly an inversion type [2], which accounts for 33%-73% of the ankle injuries [3]. After initial ankle sprain occurs, almost 35% people exacerbates to functional ankle instability (FAI) [4]. Patients with FAI have functional deficits in sports or abnormal gait kinematics [5–7]. A recent study [8] demonstrated that the use of ankle brace appeared to reduce ankle sprain recurrences and residual symptoms.

Previous kinematic studies showed that ankle brace can influence the kinematics of the foot and the ankle by changing the joint range of motion during passive ankle movement [9–11], decreasing the ankle laxity [12], and tolerating a great joint torque force [13]. Kobayashi T et al. [11] compared a semirigid ankle brace and taping on patients with chronic ankle instability (CAI) during passive ankle movement. Using a fluoroscopic image matching technique, they found that anterior translation and internal rotation in the talocrural joint remain unchanged during 20N and 20 rpm resisting passive ankle movement. The same results have been observed in the subtalar joint. Another study [12] further concluded that laxity increases significantly from pre- to postexercise. However, a brace significantly decreases ankle displacement, especially anterior ankle displacement. In terms of different designs of an ankle brace, one study [14] compared 10 types of ankle braces that significantly restrict the range of motion in all directions during passive movement and rapidly induce ankle movement compared with nonbraced conditions in patients with CAI. Furthermore, braces with stirrup design and stable/plastic reinforcement restrict inversion more effectively. Previous studies focused on passive or active ankle inversion/eversion; however, Spaulding S. J [15] studied the effect of several ankle braces on patients with FAI during normal gait and found that semirigid and soft ankle brace cannot change discrete angular and linear kinematic variables during normal walking.

Previous studies focused on the effect of ankle braces on sport or passive movement, but studies have yet to determine whether braces can improve the kinematics of walking. Ankle braces can also be commonly used for walking. For this reason, this study aims to determine the effect of a semirigid ankle brace on the gait kinematics of ankle joints through 3D-2D fluoroscopy image registration during the stance phase of walking. We hypothesize that the ankle brace would contribute to the improvement of the ankle kinematics of patients with FAI.

## 2. Material and Methods

### 2.1. Subject Characteristics

A total of 8 patients with FAI (3 males and 5 females, 10 feet) as FAI group and 10 subjects without FAI (6 males and 4 females, 10 feet) as control group were recruited (Table 1). For the FAI group, all of the following inclusion criteria [16] had to be met: (1) aged 18-40 years and BMI between 17 to 25; (2) a history of at least one ankle sprain that resulted in pain, swelling, and stiffness, prohibiting participation in sports and recreational or other activities for at least 3 weeks; (3) recurrent ankle sprain (2 or more sprains in the same ankle) or giving way (more than 2 times in the past 6 months) or feeling of instability during daily life activities in the previously injured ankle; and (4) cumberland ankle instability tool (CAIT) scores lower than 24. The control group had no history of ankle sprain in the last 2 years and a CAIT score no less than 24. Overall exclusion criteria were history of fracture or surgery in lower extremities, lower limb pain irrelated to ankle sprain, an ankle sprain in last 3 months, positive talar tilt test or anterior drawer test findings, and equilibrium deficits. All of the subjects were screened to ensure that they satisfied the inclusion and exclusion criteria. This descriptive laboratory study was approved by the Institutional Review Board of our institution, and informed consents were collected from all of the subjects.

### 2.2. Computed Tomography (CT) Scanning and Coordinate System Establishment

**Figure 1:**
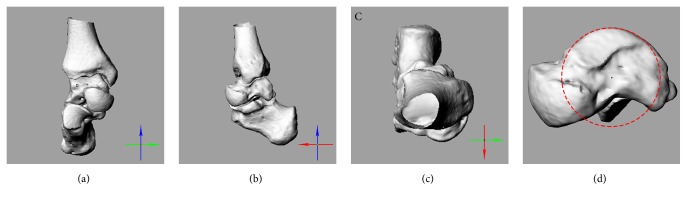
The local coordinate system of the ankle complex. The X axis (red) pointed to the anterior, Y axis (green) pointed to the left side of the object, and Z axis (blue) pointed to the proximal. (a) Anterior-posterior viewpoint; (b) medial-lateral viewpoint; (c) proximal-distal viewpoint; (d) fitting circular arc to the trochlea and its origin.

Each tested leg was subjected to CT scanning (Light Speed, GE, USA), and the scanned area covered 10 cm above the ankle joint to the bottom of the heel. The thickness and distance of each CT slice were 0.67 and 0.67mm. A loading CT scan device was used to fix the foot on a neutral position, which was defined as a 90° angle between the shank and the foot without inversion or eversion. The 3D models of the ankle complex included tibia, talus, and calcaneus were reconstructed using the AMIRA software (AMIRA, Mercury Computer Systems, Berlin, Germany, version 5.3.2). On the basis of the anatomy axis of the ankle complex [17], we established a local coordinate system (Figure 1) to calculate the six degrees of freedom (DOF) during ankle movement. The method [18] was described as follows.

Based on the talar bone anatomy [19], a transverse plane containing both apical points of the medial and lateral aspects of the trochlear tail were determined on the 3D model of the talar bone. Therefore, a series of circles perpendicular to the transverse plane and tangent to the profile of the talar dome were identified simultaneously. The lowest fitting circular arc was chosen as the sagittal plane, and the center of the circle was regarded as the origin of the coordinate system. The y axis (medial to lateral axis) was defined as the line orthogonal to the sagittal plane, passing through the origin and pointing to the left side of the object. The z axis (proximal to distal axis) was defined as the line orthogonal to the transverse plane, passing through the origin and pointing to the proximal. The x axis was created by the right-hand rule [20], which is pointing to the anterior.

The x, y, and z axes were aligned to the axes of the talus dorsiflexion/plantarflexion, inversion/eversion, and internal/external rotation, respectively, when the ankle complex was in the neutral reference position. The bone to bone angle and translation were calculated by taking the collection of the bone-axis complex as distal with respect to the proximal. The positive and negative values of a motion surrounding a specific axis were based on the right-hand rule.

### 2.3. Dual-Plane Fluoroscopic Imaging Collection

A dual-plane fluoroscopic system consisting of two same fluoroscopes (BV Pulsera, Phillips Medical, USA) arranged at a specific position and height was built, and an adjustable table was created to accommodate the X-ray beam. A custom-made platform with an embedded force plate was placed on the table to divide the gait cycle. Fluoroscopic images were collected with a pulse width of 8 ms, a resolution of 1024 × 1024, and an image intensifier diameter of 12 inches.

All the subjects maintained a walking speed of approximately 1.2 m/s on a 3.5 m long table. A valid collection of the tested leg contained one gait cycle, which started from the tested leg heel strike and ended with the toe-off. Each tested leg separately needed one valid collection in the control group with a bare foot and two valid collections in the subjects with FAI with a bare foot and a semirigid ankle brace. All the participants were trained before data were collected to ensure that they were familiar w ith the test condition. During collection, fluoroscopy 1 was positioned horizontally, and lateral views of the ankle complex were captured at 30 Hz. Fluoroscopy 2 was positioned at the same horizontal plane of fluoroscopy 1 with a 45° angle between fluoroscopy 2 and the captured medial views of the ankle complex at the same frequency.

The semirigid ankle brace (Aircast A60 Ankle Support, DJO, Europe) used in our current study was composed of nylon supporters and polyethylene lace and designed to resist inversion/eversion and internal/external rotation movements while allowing dorsiflexion/plantar flexion.

### 2.4. Image Selection and 3D to 2D Registration

Two experienced foot and ankle surgeons separately selected seven key poses of the gait phase. The image selection procedure was based on a custom-made platform with an embedded force plate, and seven key poses were chosen during one whole gait cycle. The seven selected poses were described in a previous study [21].

A 3D modeling program (Rhinoceros 5.0, Robert McNeel & Associates, Seattle, WA) was used to replicate the dual orthogonal fluoroscopic system in computer, and the semiautomatic 3D-2D registration procedure was conducted by the same surgeons. The software allowed the model to be translated and rotated in increments of less than 0.01 mm and 0.01° [22], respectively. The 3D models were considered to be matching when the model overlapped its silhouette on the fluoroscopic images as viewed from both respective virtual sources. After the matching procedure was performed, the 6DOF of the tarsal bones and the joints (i.e., talocrural and subtalar joints) could then be determined.

### 2.5. Statistical Analysis

The joint ROM of each subject during the whole stance phase in one group was defined as ROM1–ROM10. Each ROM had 6DOF results calculated as the maximum rotation/displacement of poses 1–7 minus the minimum rotation/displacement of poses 1–7. For each joint and each DOF, the mean ROM during the whole gait cycle in one group was defined as (ROM1 + ROM2 + … + ROM10)/10.

The joint position was defined as static position of the ankle joints in each of the seven poses. The sign and abbreviation of each DOF of the joint positions were defined as DF+/PF-, dorsiflexion/plantarflexion; EV+/IV-, eversion/inversion; ER+/IR-, external rotation+/internal rotation-; A+/P-, anterior/posterior displacement; L+/M-, lateral/medial displacement; P+/D-, proximal/distal displacement. The mean joint motion difference from heel strike to midstance during the stance phase was defined as the mean of (the joint positions of pose 4−the joint positions of pose 1). The mean joint motion difference from midstance to toe-off during the whole stance phase was defined as the mean of (the joint positions of pose 7−the joint positions of pose 4).

Statistical analysis was conducted using SPSS 22.0. The ROM and the joint motion differences were checked via Kolmogorov–Smirnov tests, and both of them did not meet the normal distribution criterion. Therefore, the data of two related samples of FAI without a brace and FAI with a brace were statistically analyzed using a Wilcoxon signed-rank test, and the data of the two independent samples were statistically analyzed using Mann–Whitney U test. Differences were considered statistically significant when* P*<0.05.

## 3. Result

### 3.1. Joint ROMs in Different Groups

**Figure 2:**
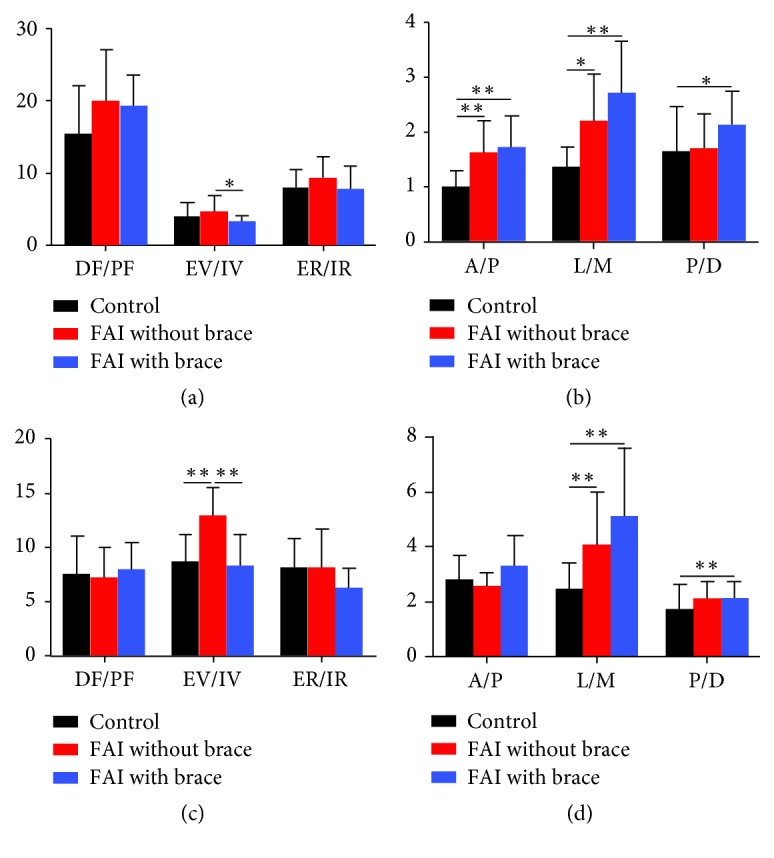
The mean range of motion of the talocrural and subtalar joints during the stance phase of walking: (a) talocrural joint rotation (°); (b) talocrural joint displacement (mm); (c) subtalar joint rotation (°); (d) subtalar joint displacement (mm) (*∗∗∗* means* P* <0.001, *∗∗* means* P* <0.01, and *∗* means* P* <0.05). DF/PF, dorsiflexion/plantarflexion; EV/IV, eversion/inversion; ER/IR, external rotation/internal rotation; A/P, anterior/posterior displacement; L/M, lateral/medial displacement; P/D, proximal/distal displacement. The error bars were standard deviations.

The mean ROMs of the talocrural joint and the subtalar joint of each group are shown in Table 2 and Figure 2.

For the talocrural joint, the ROMs in the FAI without brace group were significantly more than those in the control group in terms of lateral/medial displacement (p=0.023) and anterior/posterior displacement (p=0.005). By contrast, no significant differences were found in any of the rotation ROMs between those two groups. However, there was significantly less inversion/eversion rotation in FAI with brace group relative to FAI without brace group (p=0.037). Furthermore, the anterior/posterior (p=0.002), lateral/medial (p=0.001), and proximal/distal (p=0.041) displacements of FAI with brace group were still more than those in control group.

For the subtalar joint, the ROMs in the FAI without brace group were significantly more than those in control group in terms of eversion/inversion rotation (p=0.004) and lateral/medial displacement (p=0.001). However, there was significantly less inversion/eversion rotation in FAI with brace group relative to FAI without brace group (p=0.007). Furthermore, the lateral/medial (p=0.001) and proximal/distal (p=0.008) displacement of FAI with brace group were still more than those in control group.

### 3.2. Joint Positions in Different Groups

**Figure 3:**
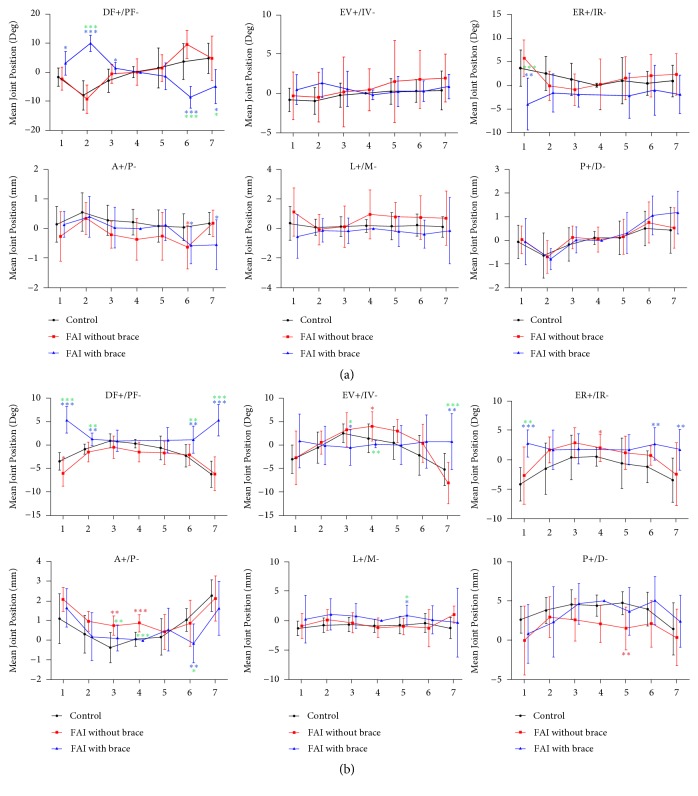
The 6DOF mean joint positions of the talocrural (a) and subtalar (b) joints during the seven poses of the stance phase. DF+/PF-, dorsiflexion/plantarflexion; EV+/IV-, eversion/inversion; ER+/IR-, external rotation+/internal rotation-; A+/P-, anterior/posterior displacement; L+/M-, lateral/medial displacement; P+/D-, proximal/distal displacement. The error bars were standard deviations (*∗* (red), comparison between FAI without brace and control; *∗* (blue), comparison between FAI with brace and control; *∗* (green), comparison between FAI without brace and FAI with brace; *∗∗∗* means* P* <0.001, *∗∗* means* P* <0.01, and *∗* means* P* <0.05).

The mean 6 DOF joint positions in seven poses are demonstrated in Figure 3. The talocrural joint of the patients in FAI with brace group was positioned significantly more dorsiflexion from pose 1 to pose 3 and more plantarflexion from pose 6 to pose 7 than those of the patients in FAI without brace or control group. The talocrural joint was also found to be significantly more internal rotation at pose 1 in FAI with brace group compared to FAI without brace or control group.

For the subtalar joint, significantly more dorsiflexion positions were detected from pose 1 to pose 2 and pose 6 to pose 7 in the FAI with brace group compared to FAI without brace or control group. And the subtalar joint was also in almost neutral position on the eversion/inversion direction. Moreover, the subtalar joint was detected less anterior in pose 3 to pose 4.

### 3.3. Joint Motion Difference in the Different Groups

**Figure 4:**
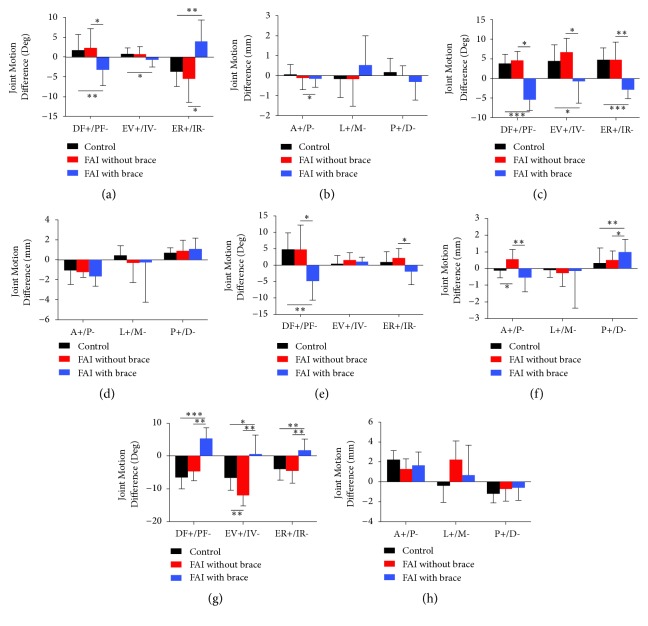
Joint motion difference of the talocrural and subtalar joints during the stance phase of walking. Heel strike to midstance: (a) talocrural joint rotation (°); (b) talocrural joint displacement (mm); (c) subtalar joint rotation (°); (d) subtalar joint displacement (mm); Midstance to Toe-off: (e) talocrural joint rotation (°); (f) talocrural joint displacement (mm); (g) subtalar joint rotation (°); (h) subtalar joint displacement (mm). DF+/PF-, dorsiflexion/plantarflexion; EV+/IV-, eversion/inversion; ER+/IR-, external rotation+/internal rotation-; A+/P-, anterior/posterior displacement; L+/M-, lateral/medial displacement; P+/D-, proximal/distal displacement. The error bars were standard deviations (*∗∗∗* means* P* <0.001, *∗∗* means* P* <0.01, and *∗* means* P* <0.05).

The mean joint motion difference from pose 1 to pose 4 and joint motion difference from pose 4 to pose 7 of the talocrural joint and the subtalar joint of each group are shown in Figure 4 and supplementary table (available here).

For the talocrural joint, there existed only the anterior/posterior joint motion difference from pose 4 to pose 7 which was statistical difference between the FAI without brace and control group (p=0.023). Statistically significant differences were observed between the FAI without brace and FAI with brace group, including joint motion difference from pose 1 to pose 4 of dorsal/plantar flexion (p=0.047), external/internal rotation (p=0.013), anterior/posterior displacement (p=0.021), joint motion difference from pose 4 to pose 7 of dorsal/plantar flexion (p=0.037), external/internal rotation (p=0.047), anterior/posterior displacement (p=0.005), and proximal/distal displacement (p=0.037). Furthermore, there was still difference between the FAI with brace and control group, including joint motion difference from pose 1 to pose 4 in all of the three rotations (dorsal/plantar flexion (p=0.019), eversion/inversion (p=0.049), and external/internal rotation (p=0.004)) and joint motion difference from pose 4 to pose 7 in dorsal/plantar flexion (p=0.004) as well as proximal/distal displacement (p=0.049).

For the subtalar joint, the joint motion difference between FAI without brace and control group was not significant. The comparison between FAI without brace and FAI with brace group showed that the joint motion difference from pose 1 to pose 4 and joint motion difference from pose 4 to pose 7 of dorsal/plantar flexion, external/internal rotation, and inversion/eversion rotation were significant. Similarly, those differences between FAI without brace and FAI with brace group also existed between FAI with brace and control group. Except the talocrural joint anterior/posterior displacement, all of the statistically different results of the joint motion difference had an adverse sign between the FAI without brace and FAI with brace group.

## 4. Discussion

The use of ankle brace for FAI is beneficial clinically. Previous studies showed that ankle brace can reduce the large inversion range of motion causing injury due to lateral ankle sprain [23], without impeding functional performance and reducing muscle activity [24], consequently improving the proprioception of ankle joint [25]. Therefore, the normalization of the abnormal joint may be a key mechanism to prevent an initial lateral ankle sprain. Many studies [26–28] have indicated that ankle brace can effectively limit the mechanically imposed ankle inversion when ankle is in the position where lateral ankle sprain usually occurs. In terms of gait kinematics, one study [15] focused on the influence of ankle brace on gait. Several studies [11] have separately evaluated the dynamic kinematics of the talocrural and subtalar joints. Our study aimed to indicate the effect of a semirigid ankle brace on the kinematics of talocrural and subtalar joints during gait.

Our study presented further findings based on previous studies. Comparing with the people without FAI, the patients with FAI had more instability without brace, referred to increase in 6DOF of the movement between two bones, in the lateral/medial, anterior/posterior displacement of the talocrural joint, the eversion/inversion of the subtalar joint, and the lateral/medial displacement during gait. For the joint position, the patients with FAI without a brace had more anterior and inverted position of the subtalar joint from the midstance to the toe-off phase of the gait cycle. Several contradictory findings from previous studies were obtained. Monaghan K [29] found that subjects with CAI are significantly more inverted in the frontal plane than the controls during the early stance of gait. However, De Ridder R [30] showed that patients with CAI have a more everted foot position than the control group during walking. Considering the low accuracy of traditional camera-optoelectronic systems and their inability to study talocrural and subtalar joints separately, we concluded that our current study had more precise and updated results than previous studies. Radiographic shape matching techniques (3D-2D model-image registration) have been used to evaluate joint kinematics, especially on ankle motion analysis during dorsiflexion-plantar flexion activities and quasistatic gait, indicating that more precise results may be found, and conclusions presented in other studies should be reexamined.

A semirigid ankle brace could significantly restrict the inversion/eversion ROM of talocrural and subtalar joints. However, the ROM of most of the displaced talocrural and subtalar joints still increased compared with that of the control group. The ankle brace altered the position of the ankle joints during the stance phase of walking. More dorsiflexion from heel strike to midstance, more plantarflexion from midstance to toe-off of the talocrural joint, and more dorsiflexion and less eversion or inversion in the whole stance phase were detected in our study. The result of the joint motion difference was indicated during walking. The talocrural joint of patients with FAI under the brace condition had more movement in the plantarflexion and posterior directions in the whole stance phase, more movement in the external rotation direction from heel strike to midstance, and more internal rotation and proximal direction movement from midstance to toe-off. For the subtalar joint, our result indicated more plantarflexion, inversion, and inversion movement from heel strike to midstance and more dorsiflexion, eversion, and external rotation movement from midstance to toe-off. More significant results were observed in the joint motion difference between the patients with FAI in the brace group and the control group than those between the patients with FAI with and without the brace. This finding suggested the partial hypercorrection of the semirigid ankle brace. Hypercorrections mostly appeared during rotation, suggesting that the ankle brace mainly altered the ankle joint rotation. Since there was only one study [15] focused on the effect of ankle brace on gait kinematics of patients with FAI by using traditional camera-optoelectronic systems and indicated ankle brace does not change any gait parameters. Our study might provide a new mechanism of ankle braces.

The ankle brace was used by the patients with FAI in our current study to restrict the excessive inversion/eversion ROM of the talocrural and subtalar joints during gait. No previous studies indicated this role of the ankle brace. To our knowledge, this study is the first to consider the effects of external ankle support on the gait kinematics of the talocrural and subtalar joint kinematics through 3D-2D registration. These findings might support the use of ankle brace for patients with FAI to possibly increase the stability of the ankle joint complex during daily walking. The ankle brace resulted in the alteration of the movement of the talocrural and subtalar joints in the opposite direction on several DOF during the partial or whole stance phase, and this overcorrection might be a key mechanism of ankle braces. A previous study [31] that the increased touchdown plantar flexion may be the mechanism that causes ankles with a history of ankle sprains to have an increased susceptibility to subsequent sprains. Coincidentally, in our study, the talocrural and subtalar joints were placed in a more dorsiflexion position from heel strike to midstance after the patients wore the ankle brace in our study. These factors constituted the mechanism of the semirigid ankle brace.

Some methodological limitations should be considered. In terms of the low number of fluoroscopy frames of the dual-plane fluoroscopic system, we had to decompose the whole gait cycle into seven key poses. The 3D-2D registration procedure was semiautomatic; that is, ample time was necessary to accomplish image matching. This procedure resulted in a relatively small sample size of this study. Aircast A60, a common semirigid ankle brace, was chosen as the object of this study. As such, our current study might have some discrepancies compared with other related studies on braces because of the different structures of ankle braces.

## 5. Conclusion

The proposed semirigid ankle brace could normalize the inversion/eversion ROM of the talocrural and subtalar joints of patients with FAI, change the joint position in several poses, and partly alter the movement of the talocrural and subtalar joints of patients with FAI in opposite directions.

## Figures and Tables

**Table 1:** Subject characteristics.

	Control	FAI
Number of subjects	10	8
Number of feet	10	10
Gender	6M/4F	3M/5F
Age (years)	25.2±1.8	22.4±1.6
BMI (kg/m^2^)	21.5±1.4	20.6±1.7
CAIT scores	29.7±0.9	16.3±4.1

FAI: functional ankle instability; BMI: body mass index; CAIT scores: Cumberland ankle instability tool scores.

Mean values and their SD of age, BMI, and CAIT scores are listed in Table 1.

**Table 2:** Range of motion of the talocrural and subtalar joints during the stance phase of walking.

	ROM											
	Talocrural Joint						Subtalar Joint					
	Rotation (°)			Displacement (mm)			Rotation (°)			Displacement (mm)		
	DF/PF	EV/IV	ER/IR	A/P	L/M	P/D	DF/PF	EV/IV	ER/IR	A/P	L/M	P/D
Control	15.38±6.72	3.99±1.96	8.00±2.52	1.00±0.30	1.37±0.37	1.65±0.81	7.60±3.49	8.76±2.43	8.14±2.65	2.83±0.87	2.47±0.95	1.74±0.90
FAI without brace	19.89±7.19	4.72±2.19	9.26±2.98	1.62±0.58	2.20±0.85	1.71±0.63	7.23±2.80	12.98±2.53	8.18±3.54	2.56±0.52	4.06±1.95	2.12±0.62
FAI with brace	19.34±4.20	3.31±0.78	7.65±3.32	1.72±0.58	2.71±0.94	1.79±0.60	7.97±2.51	8.33±2.92	6.27±1.82	3.31±1.10	5.12±2.48	2.13±0.61

DF/PF, dorsiflexion/plantarflexion; EV/IV, eversion/inversion; ER/IR, external rotation/internal rotation; A/P, Anterior/posterior displacement; L/M, lateral/medial displacement; P/D, proximal/distal displacement.

Mean ROM and their SD were listed in Table 2.

## Data Availability

The datasets supporting the conclusions of this article are included within the article and its supplementary materials.

## Supplementary Materials

Supplementary Materials Tables 3 and 4 were joint motion difference of the talocrural and subtalar joints during the gait cycle, which were corresponding to Figure 4 in the manuscript. Seven key poses of gait cycle (from the first line starts at the left to the second line ends at the right) are pose 1-pose 7 in turn. The gait cycle was determined as the heel strike (pose 1) and the toe-off (pose 7) were identified as the emergence and disappearance of the force under the tested foot. The foot flat (pose 2) was identified as the emergence of the force under the first metatarsal region of the tested foot. The toe-off of the contra-lateral side (pose 3) was identified as the pose before the disappearance of the force under the contra-lateral foot. The midstance (pose 4) was identified as the midterm of the single leg stance phase. The heel-off of the tested side (pose 5) was identified as the disappearance of the force under the heel region of the tested foot. The heel strike of the contra-lateral side (pose 6) was identified as the emergence of the force under the contra-lateral foot.
