# Supplementary material for: Effect of a Semirigid Ankle Brace on the In Vivo Kinematics of Patients with Functional Ankle Instability during the Stance Phase of Walking
Source: Biomed Res Int. 2019 Apr 8;2019:4398469. doi: 10.1155/2019/4398469 (PMC6476145; doi:10.1155/2019/4398469)

Table 3 Joint Motion Difference of the Talocrural and Subtalar Joints from Heel-strike to Mid-stance during the Stance Phase of Walking

	Joint Motion Difference 1											
	Talocrural Joint						Subtalar Joint					
	Rotation (°)			Displacement (mm)			Rotation (°)			Displacement (mm)		
	DF+/PF-	EV+/IV-	ER+/IR-	A+/P-	L+/M-	P+/D-	DF+/PF-	EV+/IV-	ER+/IR-	A+/P-	L+/M-	P+/D-
Normal	1.74±4.03	3.99±1.96	8.00±2.52	0.07±0.50	-0.16±0.93	0.17±0.70	3.76±2.43	4.43±4.15	4.69±3.09	-1.05±1.43	0.42±1.01	0.71±0.50
FAI	2.29±4.94	0.76±1.95	9.26±2.98	-0.10±0.61	-0.16±1.35	0.01±0.50	4.55±2.29	6.63±3.57	4.72±4.56	-1.21±0.55	-0.29±1.98	0.85±1.09
FAI with Brace	-3.18±4.06	-0.67±1.86	3.98±5.49	-0.14±0.43	0.53±1.48	-0.30±0.93	-5.34±2.81	-0.68±5.60	-2.77±2.33	-1.65±0.99	-0.25±4.01	1.06±1.14

DF/PF, dorsiflexion/plantarflexion; EV/IV, eversion/inversion; ER/IR, external rotation/internal rotation; A/P, Anterior/posterior displacement; L/M, lateral/medial displacement; P/D, proximal/distal displacement.

Table 4 Joint Motion Difference of the Talocrural and Subtalar Joints from Mid-stance to Toe-off during the Stance Phase of Walking

	Joint Motion Difference 2											
	Talocrural Joint						Subtalar Joint					
	Rotation (°)			Displacement (mm)			Rotation (°)			Displacement (mm)		
	DF+/PF-	EV+/IV-	ER+/IR-	A+/P-	L+/M-	P+/D-	DF+/PF-	EV+/IV-	ER+/IR-	A+/P-	L+/M-	P+/D-
Normal	4.75±5.09	0.38±2.51	0.92±3.23	-0.11±0.45	-0.08±0.45	0.33±0.92	-6.49±3.55	-6.58±3.94	-3.96±3.35	2.22±0.92	-0.37±1.64	-1.18±0.92
FAI	4.74±7.45	1.48±2.31	2.10±2.98	0.55±0.60	-0.26±0.81	0.49±0.57	-4.65±2.95	-11.96±3.19	-4.50±3.81	1.26±1.04	2.22±1.89	-0.69±1.24
FAI with Brace	-4.82±5.86	1.06±1.40	-1.90±4.09	-0.55±0.85	-0.13±2.24	0.99±0.78	5.29±3.37	0.54±5.86	1.72±3.46	1.63±1.38	0.68±3.01	-0.57±1.27

DF/PF, dorsiflexion/plantarflexion; EV/IV, eversion/inversion; ER/IR, external rotation/internal rotation; A/P, Anterior/posterior displacement; L/M, lateral/medial displacement; P/D, proximal/distal displacement.

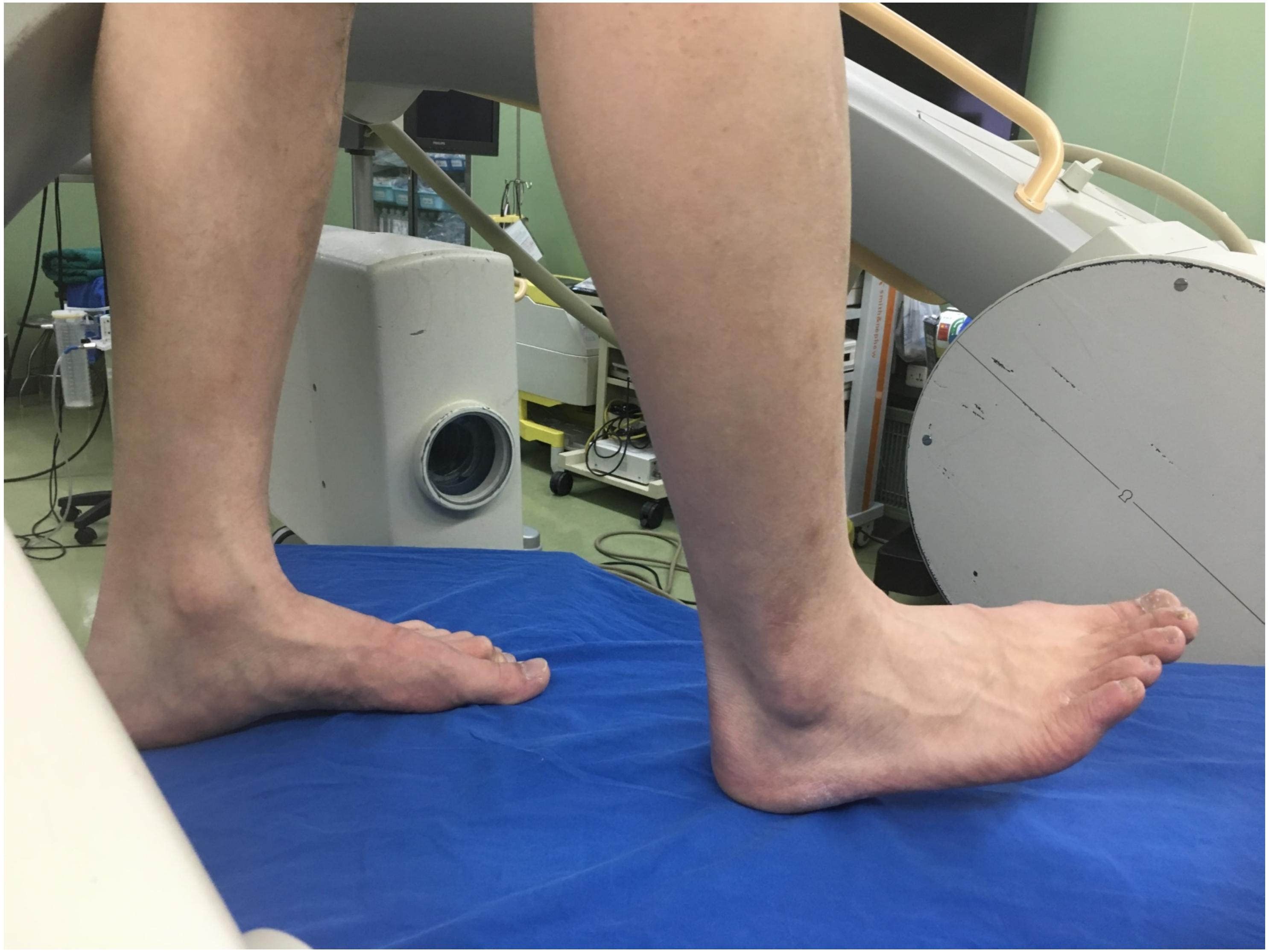

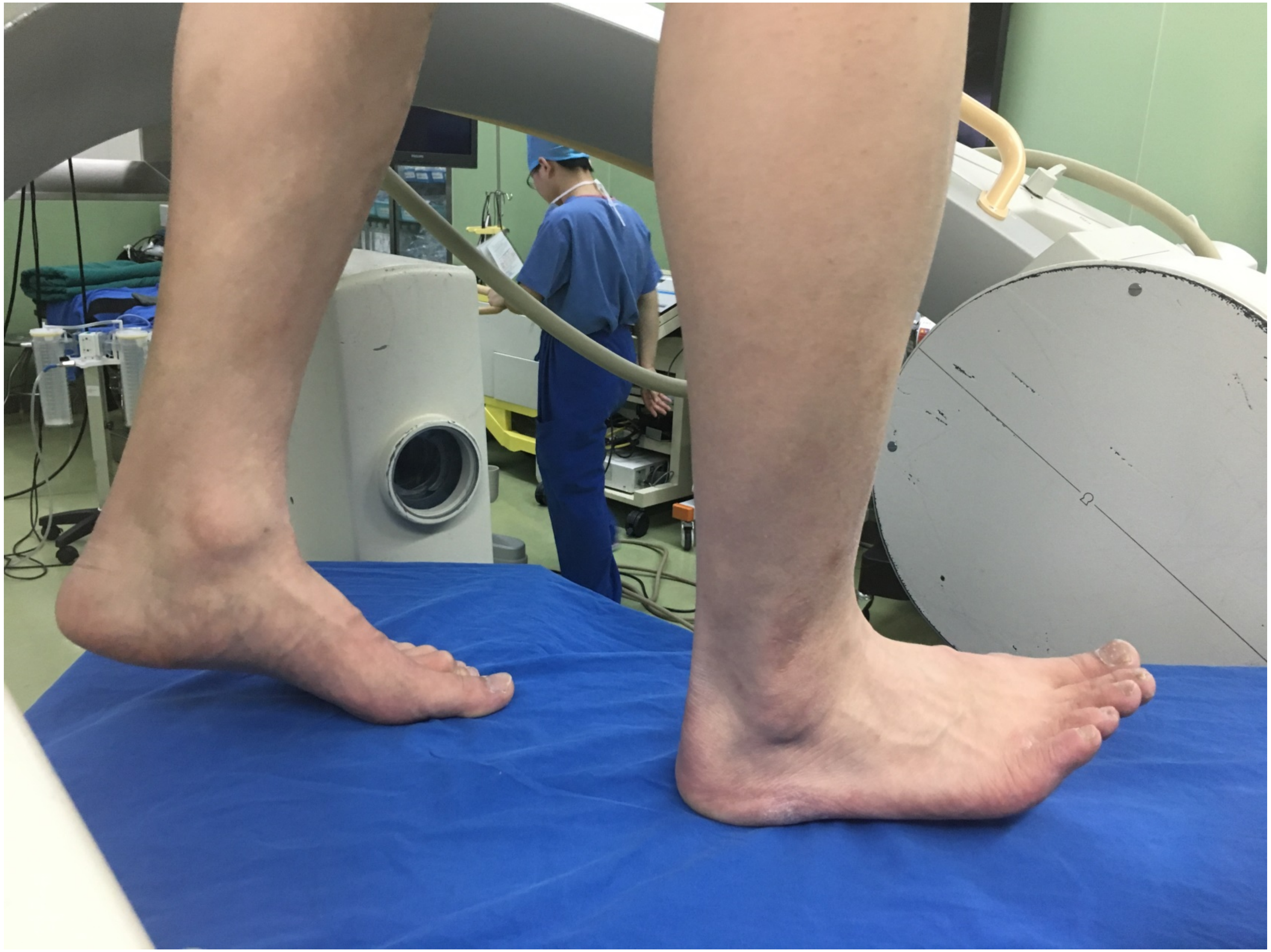

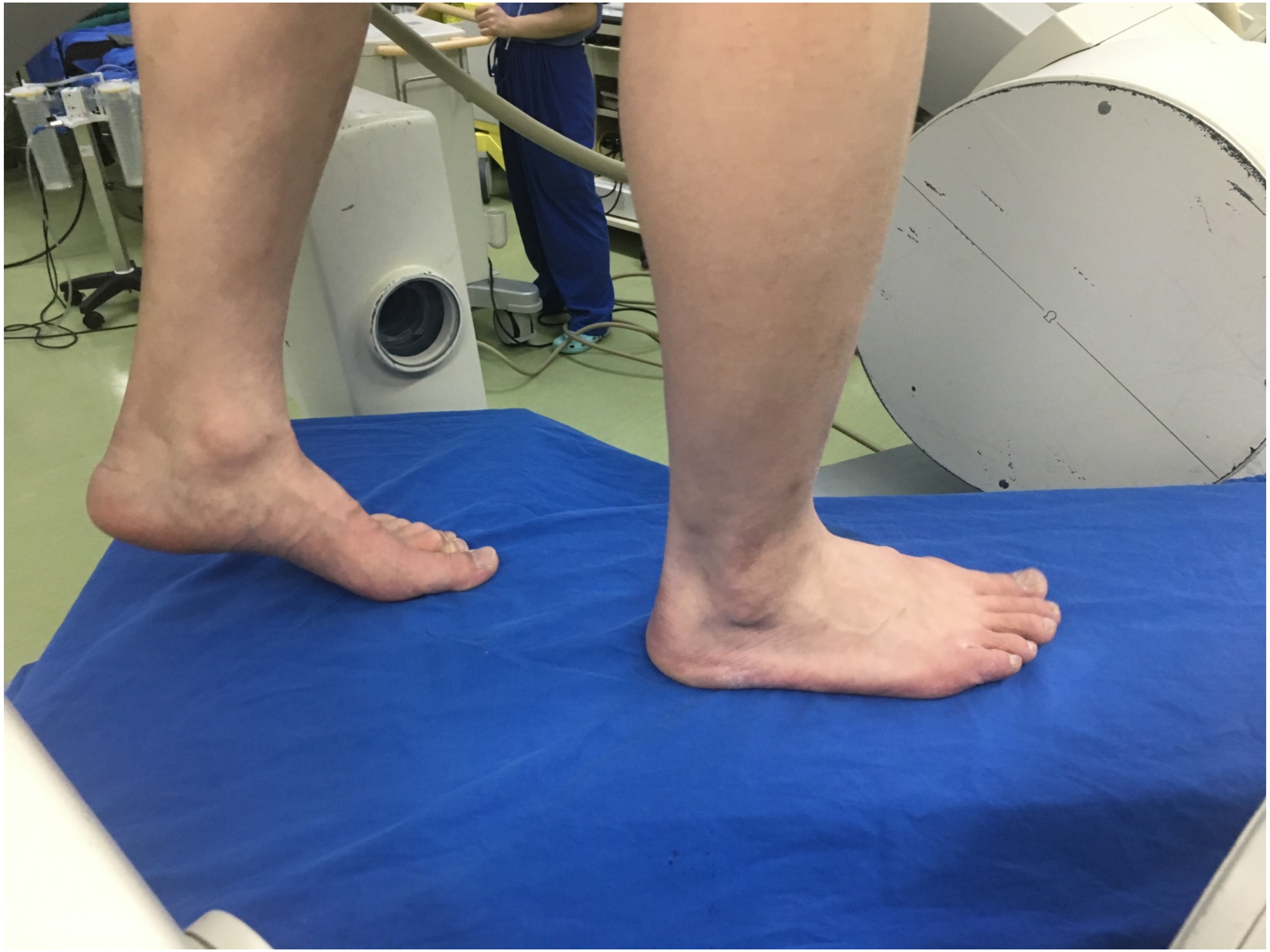

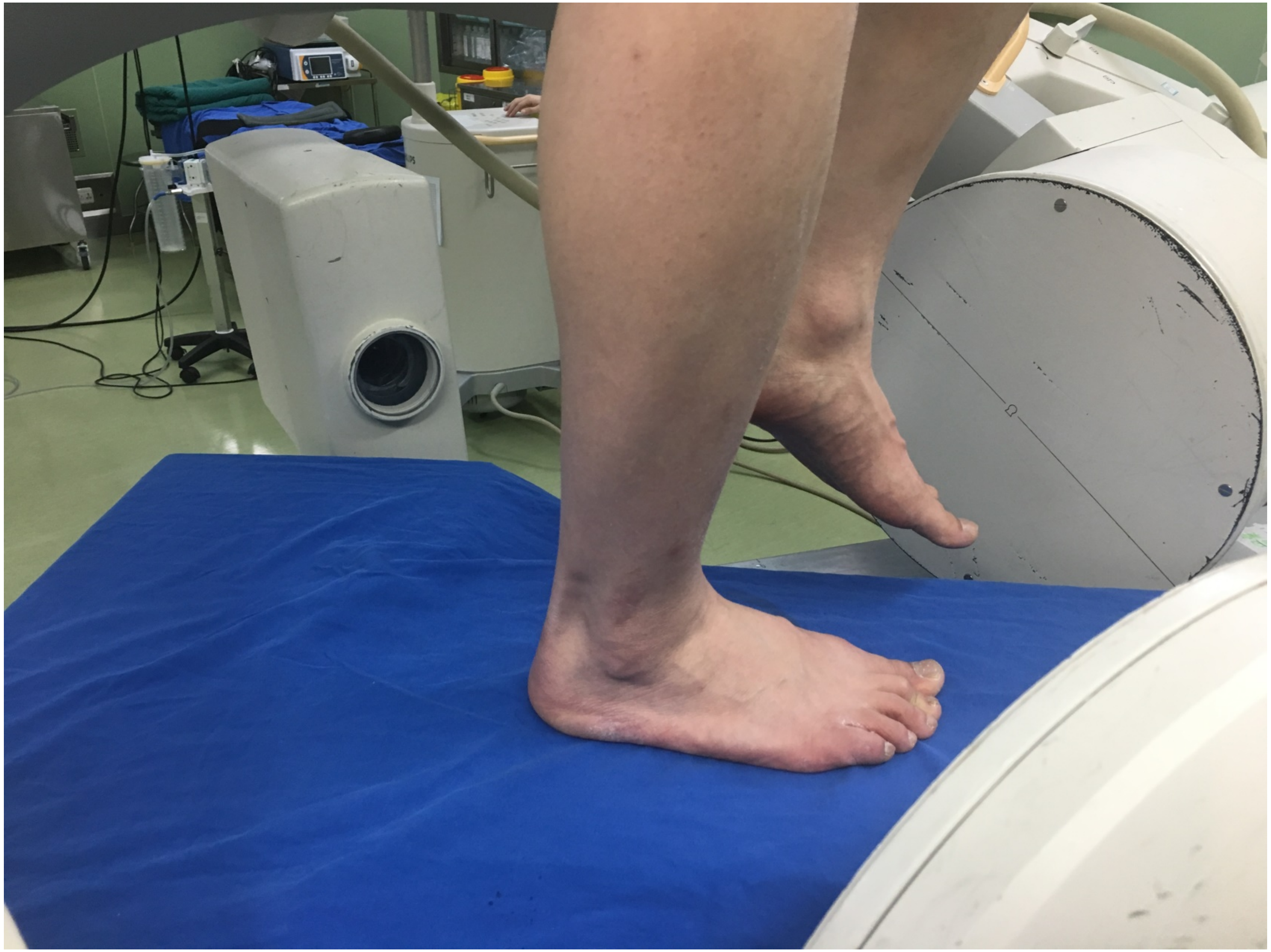

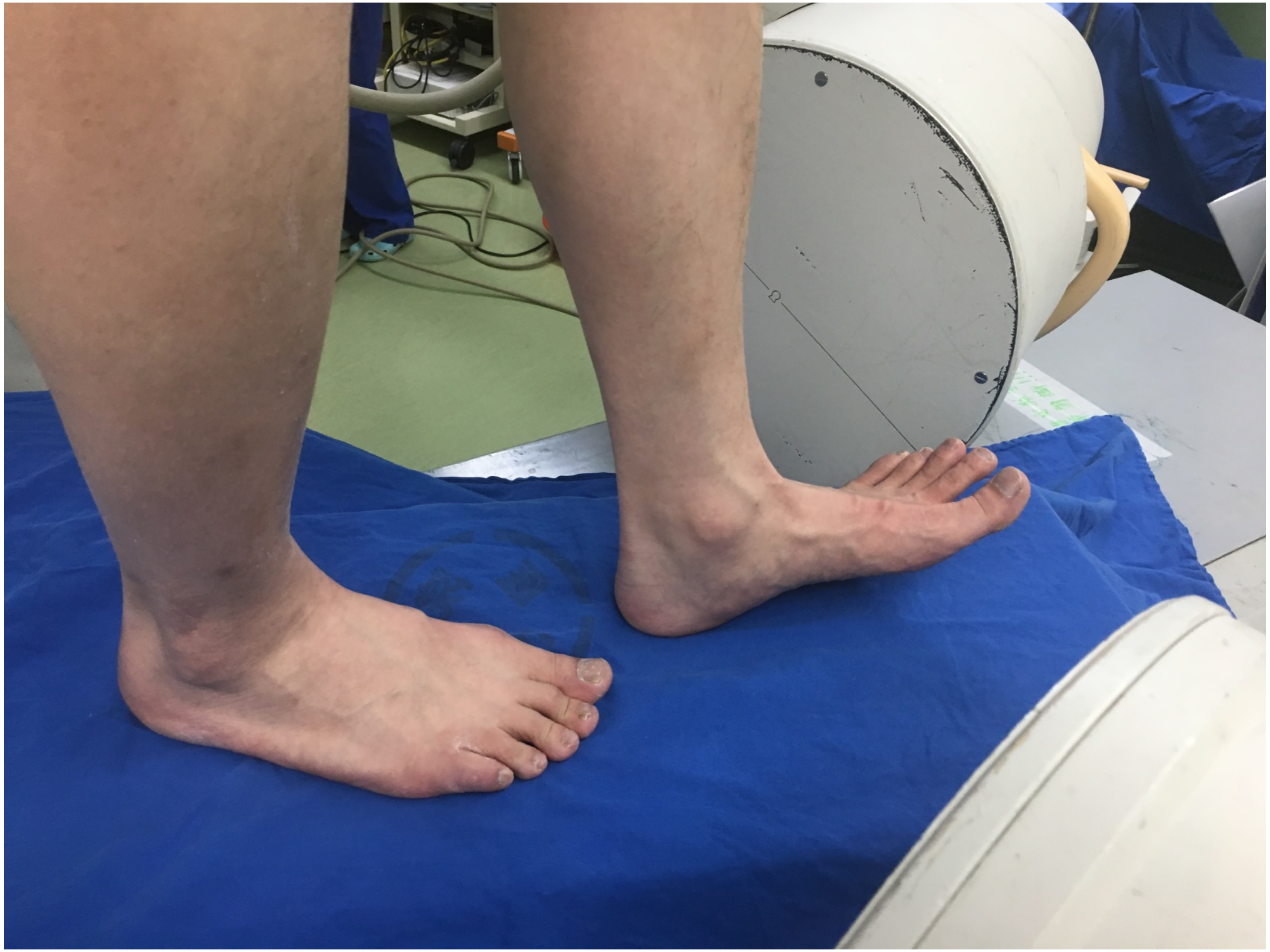

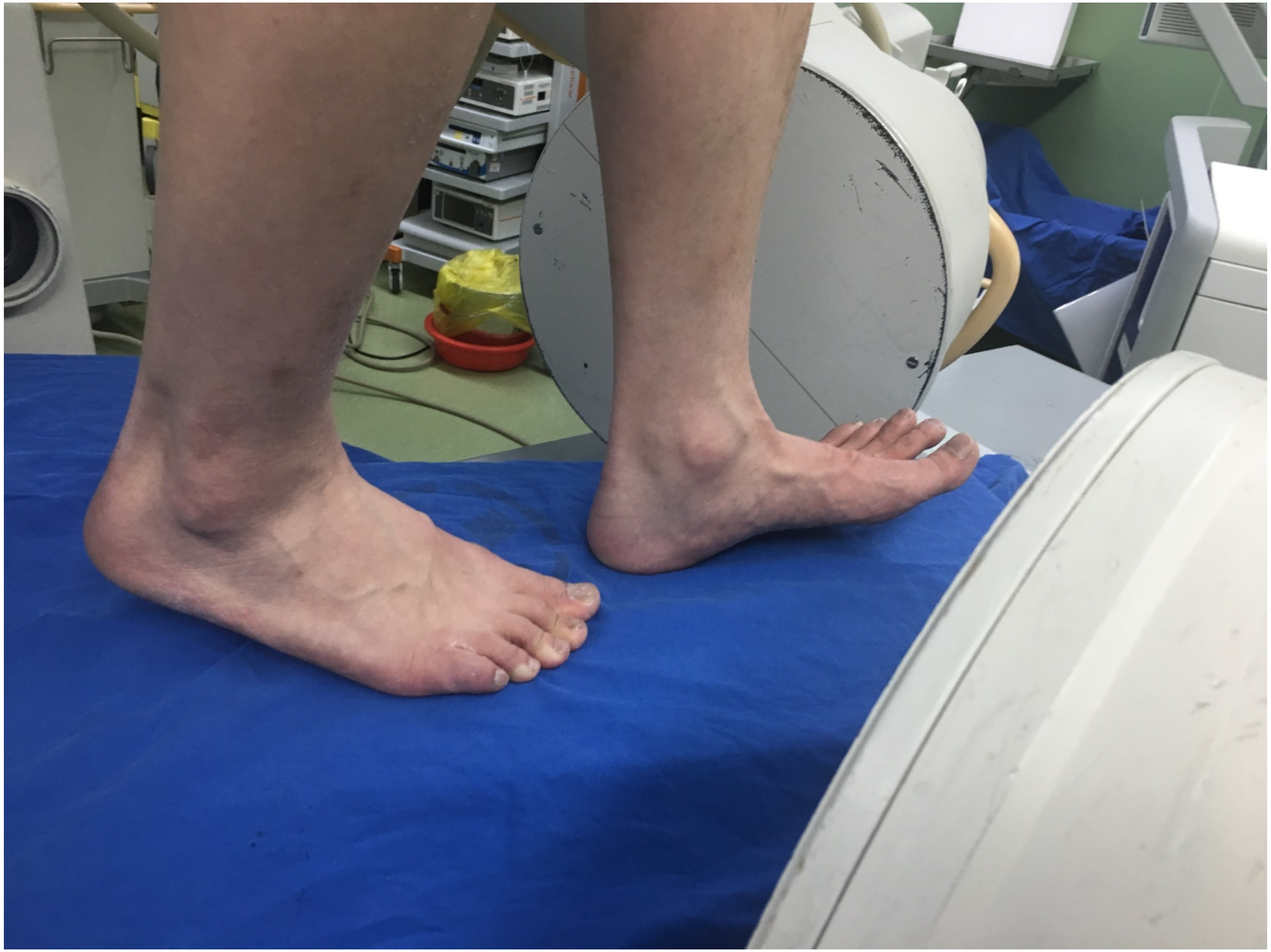

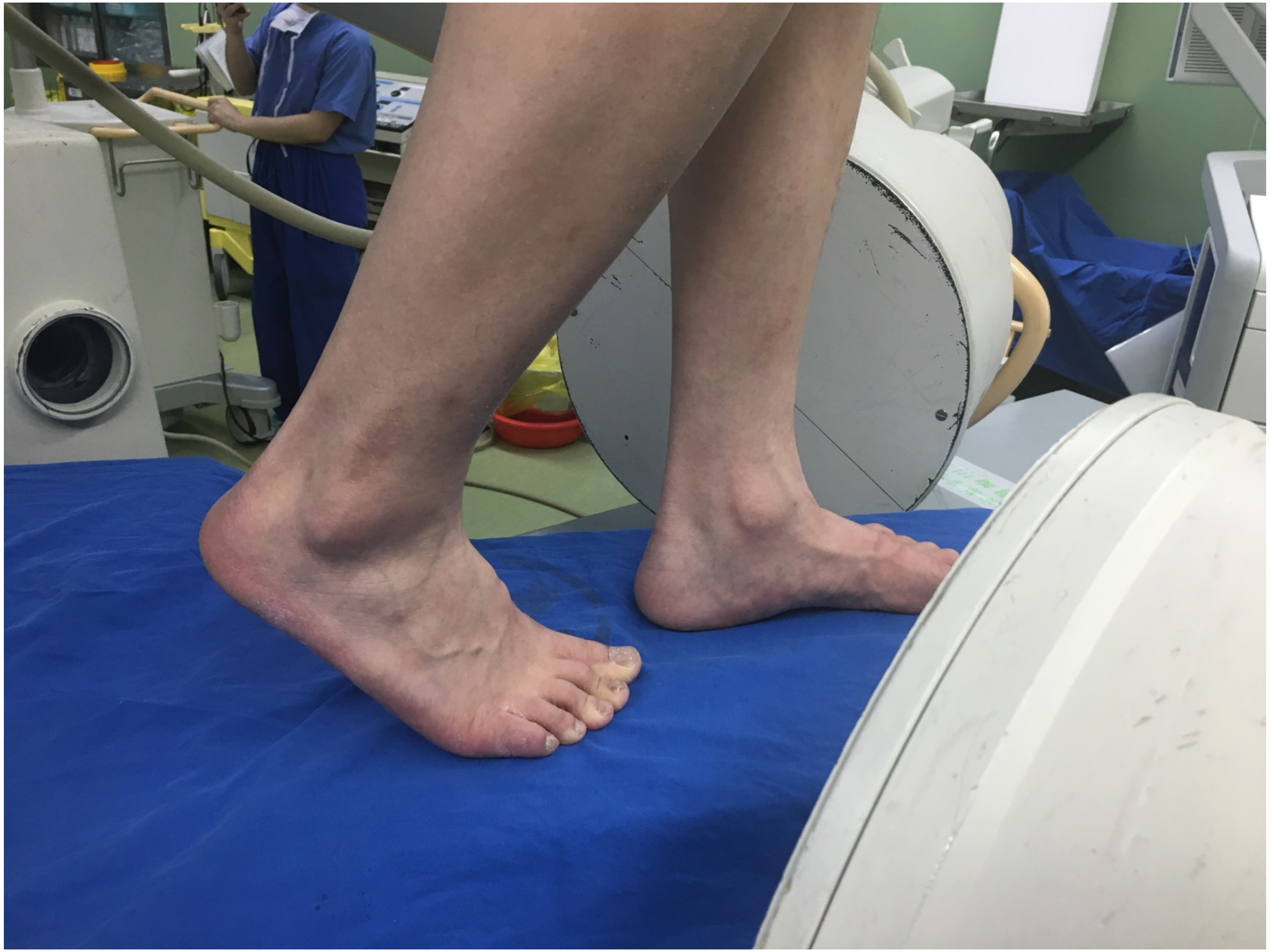

Supplement: Supplementary Materials — Tables 3 and 4 were joint motion difference of the talocrural and subtalar joints during the gait cycle, which were corresponding to Figure 4 in the manuscript. Seven key poses of gait cycle (from the first line starts at the left to the second line ends at the right) are pose 1-pose 7 in turn. The gait cycle was determined as the heel strike (pose 1) and the toe-off (pose 7) were identified as the emergence and disappearance of the force under the tested foot. The foot flat (pose 2) was identified as the emergence of the force under the first metatarsal region of the tested foot. The toe-off of the contra-lateral side (pose 3) was identified as the pose before the disappearance of the force under the contra-lateral foot. The midstance (pose 4) was identified as the midterm of the single leg stance phase. The heel-off of the tested side (pose 5) was identified as the disappearance of the force under the heel region of the tested foot. The heel strike of the contra-lateral side (pose 6) was identified as the emergence of the force under the contra-lateral foot. [file 4398469.f1.pdf]
